# Clinical Characteristics of Patients with Pustular Psoriasis: A Single-Center Retrospective Observational Study

**DOI:** 10.3390/vaccines10081171

**Published:** 2022-07-23

**Authors:** Paolo Gisondi, Francesco Bellinato, Giampiero Girolomoni

**Affiliations:** Department of Medicine, Section of Dermatology and Venereology, University of Verona, 37126 Verona, Italy; paolo.gisondi@univr.it (P.G.); giampiero.girolomoni@univr.it (G.G.)

**Keywords:** psoriasis, pustular psoriasis, generalized pustular psoriasis, palmoplantar pustulosis, acrodermatitis continua of Hallopeau, von Zumbush

## Abstract

Clinical and epidemiologic data on pustular psoriasis are scarce. To investigate the phenotypes of pustular psoriasis and the patients’ characteristics observed in a real-life retrospective observational study. The number of incident cases of pustular psoriasis registered in the period 2005–2021 was retrieved from the electronic medical records of the University Hospital of Verona. One hundred and forty cases of pustular psoriasis were collected. Ninety-one out of 140 patients (65%) were females, with a median (IQR) age of 57 (43–66) years. According to the ERASPEN classification criteria, 116 patients (83%) had palmoplantar pustulosis (PPP), 13 (9%) generalized pustular psoriasis (GPP), and 11 (8%) acrodermatitis continua of Hallopeau (ACH). Gender distribution and median age were consistent among the three groups. The prevalence of psoriatic arthritis in GPP, ACH, and PPP was 8%, 36%, and 28%, respectively. During the same period, a total of 4718 cases of plaque psoriasis were retrieved, with a 1:34 ratio of pustular over plaque psoriasis. Pustular psoriasis is much rarer than plaque psoriasis, with PPP being the more common subtype.

## 1. Introduction

Pustular psoriasis comprises a group of inflammatory cutaneous diseases characterized by multiple non-follicular pustules over an erythematous base corresponding histologically to collections of neutrophils in the epidermis [1]. Pustular psoriasis is genetically and clinically separate from psoriasis vulgaris, even if previous or concomitant plaque psoriasis may be found. The identification of specific genetic mutations (i.e., IL36RN, CARD14, and AP1S3) in subgroups of patients suggest the role of the genetic component and a dysregulation in different pathways in the pathogenesis [2,3]. Historically, different entities of pustular psoriasis have been characterized with respect to their onset and morphologic pattern including acute (von Zumbusch), subacute annular, chronic (acral), and mixed [4]. The European Rare and Severe Psoriasis Expert Network (ERASPEN) recognized three main clinical phenotypes of pustular psoriasis, namely Generalized Pustular Psoriasis (GPP), Acrodermatitis Continua of Hallopeau (ACH), and Palmoplantar Pustulosis (PPP) [5]. GPP, the most severe variant, is a multisystemic inflammatory disease characterized by intermittent flares of an extensive skin erythema covered by aseptic pustules, systemic symptoms (i.e., fever and fatigue), and extracutaneous manifestations (i.e., arthritis, uveitis, acute respiratory distress syndrome, and cardiovascular shock, cholestasis). ACH is characterized by pustular lesions involving acral extremities of the hands and feet, with onychodystrophy evolving to progressive destruction of the nail apparatus (anonychia), with or without underlying osteolysis. PPP is the most common variant, presenting with sterile, erupting pustules, located symmetrically on the palms and soles, with a chronic and cyclic course, occurring on a desquamative and erythematous background. A predilection for histologic involvement of the acrosyringium is a specific feature of PPP.

Few epidemiological studies have been conducted on pustular psoriasis, mostly focused on GPP and in Japan, whereas epidemiologic data in European countries are rarer [6]. The aim of this study was to investigate the phenotypes of pustular psoriasis and the patients’ characteristics observed in a real-life setting.

## 2. Methods

This is a retrospective observational study in which the number of incident cases of pustular psoriasis diagnosed in the period 2005–2021 was retrieved and the main patient characteristics were described. Moreover, the number of incident cases of pustular psoriasis was compared to that of plaque psoriasis observed in the same period. All pustular and plaque psoriasis cases were retrospectively derived from the electronic database of the Dermatology Division of University Hospital of Verona (Italy) over the same time period.

The cases of pustular psoriasis were classified according to ERASPEN criteria [5]. GPP was defined as macroscopically visible primary sterile pustules occurring on non-acral skin and not within psoriasis plaques. GPP is diagnosed when the condition has relapsed at least once or when it persisted for more than 3 months. Additionally, a drug reaction such as acute generalized exanthematous pustulosis (AGEP) has been ruled out. GPP can occur with or without systemic inflammation; for systemic inflammation, the American Society of Chest Physicians’ definition of fever >38 °C and leucocytosis (WBC > 12 × 10^9^/L) was considered [7]. PPP was defined as primary, persistent (>3 months), sterile, macroscopically visible pustules on the palms and/or soles; ACH was defined as primary, persistent (>3 months), sterile, macroscopically visible pustules affecting the nail apparatus (although it could be not restricted to it) [5]. GPP, PPP, and ACH could occur with or without plaque psoriasis. The diagnosis of pustular and plaque psoriasis was made by a dermatologist on a clinical base and confirmed by histological examination when needed.

## 3. Statistical Analysis

All of the incident cases of pustular and chronic plaque psoriasis diagnosed and registered in the period 2005–2021 were retrieved from a Microsoft Access electronic database (v2000; Microsoft Corp., Redmond, WA, USA). Search terms included the following: “Psoriasis”, “Pustular psoriasis”, “Plaque psoriasis” “Pustulosis of Palms and Soles”, “Pustulosis Palmaris et Plantaris”, “Palmoplantaris Pustulosis”, “Pustular Psoriasis of Palms and Soles”, “Generalized Pustular Psoriasis”, “von Zumbush”, “Acrodermatitis Continua of Hallopeau”, “Acropustolosis”, and “Subungual pustulosis”. Each case was analyzed, and the following data were collected: age, gender, phenotypes of pustular psoriasis, psoriatic arthritis, smoking habit, and body mass index (BMI). Body temperature and white blood cell count was considered in patients with GPP. In the descriptive analysis, results were presented as the median value and interquartile range (IQR) or proportion when appropriate. Fisher’s exact test and the Kruskal–Wallis test were used to compare categorical and unpaired non-normally distributed quantitative data, respectively. Moreover, the ratio of the number of incident cases of pustular over plaque psoriasis made in the same period of observation was calculated. Statistical analyses were performed using Stata v13 (Stata Corp., College Station, TX, USA).

## 4. Results

**Figure 1:**
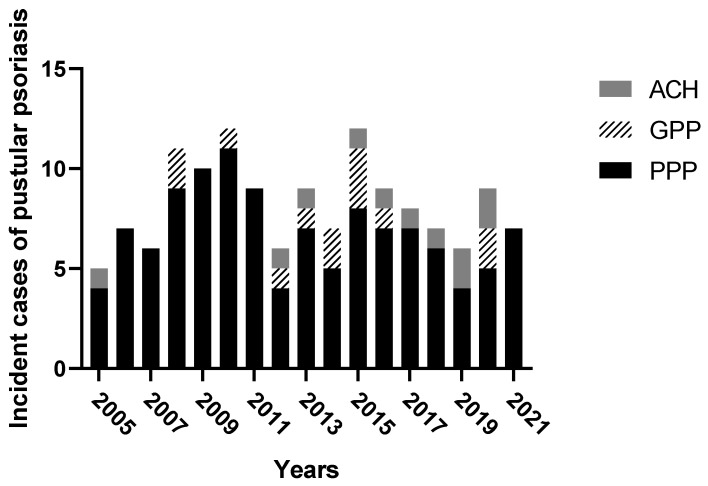
The number of incident cases of pustular psoriasis diagnosed from 2005 to 2021. Palmoplantar psoriasis (black histograms), generalized pustular psoriasis (dashed histograms), and acrodermatitis continua of Hallopeau (grey histograms).

**Figure 2:**
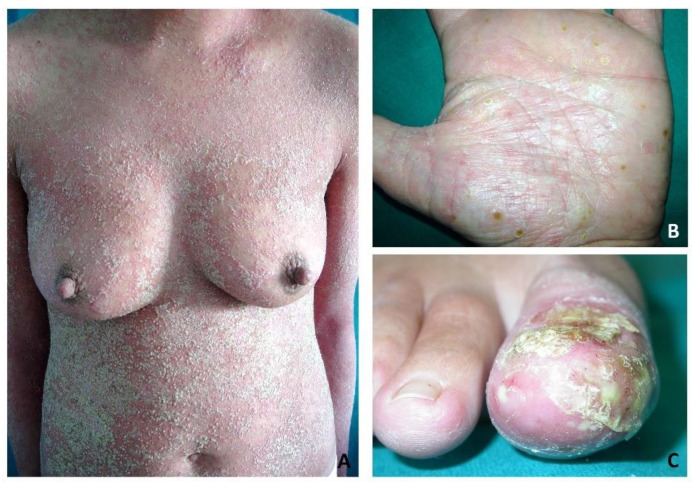
Clinical presentation of three forms of pustular psoriasis. Generalized pustular psoriasis showing diffuse erythroderma covered by confluent pustules coalescing into pustular lakes (**A**). Palmoplantar pustulosis showing pustular lesions involving the palmar area (**B**). Typical lesions of acrodermatitis continua of Hallopeau involving the toe and leading to the destruction of the nail apparatus (**C**).

**Figure 3:**
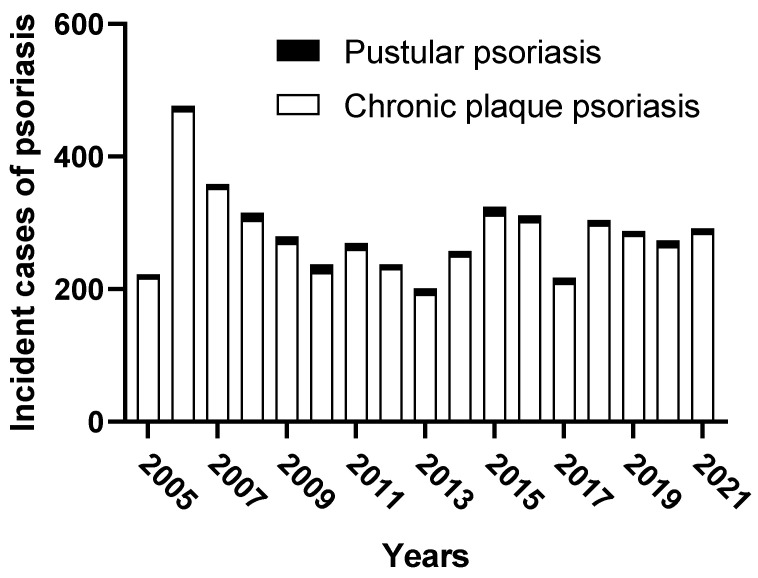
The number of incident cases of plaque versus pustular psoriasis diagnosed per year from 2005 to 2021. Plaque psoriasis (white histograms), pustular psoriasis (black histograms).

A total of 140 cases of pustular psoriasis were retrieved. The absolute number of incident cases of pustular psoriasis diagnosed from 2005 to 2021 is shown in Figure 1, ranging from five in 2005 to 12 in 2015. Ninety-one out of 140 patients (i.e., 65%) were females, the median (IQR) age was 57 (43–66) years, and the median BMI (IQR) was 26 (23–32). According to the ERASPEN classification criteria, 116 patients (83%) had PPP, 13 (9%) GPP, and 11 (8%) ACH (Table 1, Figure 2). Gender distribution and median age were consistent among the three groups. Thirty-eight out of 140 patients (27%) had concomitant PsA. In particular, 30 had peripheral arthritis (median number of four swollen and six tender joints), four patients had dactylitis, three enthesitis, and one patient had axial involvement. The prevalence of PsA in GPP, ACH, and PPP was 1 (8%), 4 (36%), and 33 (28%), respectively. The prevalence of smokers in PPP, ACH, and GPP was 82 (39%), 4 (36%), and 5 (71%), respectively. Concomitant erythematous squamous plaques were found in 23 (20%), 2 (18%), and 4 (31%) of those with PPP, ACH, and GPP, respectively. In patients with PPP, pustules were localized on both the palms and soles in 68 (59%) of cases, only on the palms in 9 (8%), and only on the soles in 39 (33%). Interestingly, the palms and soles were spared in those with GPP. Fingernails were affected in 9 out of 11 cases of ACH, with a median of 3 digits involved. Systemic inflammation (i.e., fever > 38 °C and leucocytosis) was found in 3 out of 13 patients with GPP. Skin biopsy for histologic diagnosis was required in 24 patients, in 13 patients with GPP, 11 patients with PPP, and none with ACH. The absolute number of incident cases of plaque versus pustular psoriasis diagnosed per year is shown in Figure 3. The ratio of new diagnoses of pustular versus over plaque psoriasis made in the same period of observation was 1: 33.7, ranging from 1:18.8 in 2010 to 1:67.0 in 2006.

## 5. Discussion

The main finding of the study is that the number of cases of pustular psoriasis observed over 17 years was relatively small, particularly when compared to those of plaque psoriasis, being 1:34 the ratio of pustular over plaque psoriasis. The University Hospital of Verona is the main public hospital of the city, serving a population of about one million inhabitants and the division of Dermatology represents a regional and extra-regional referral center treatment of psoriasis, thus it is unlikely that there have been a relevant number of pustular psoriasis cases that have escaped our analysis. We found that pustular psoriasis was more common in females (65%) than in males. This is consistent with several Asian studies, whereas this finding is controversial in other Western countries [4,7,8,9,10,11,12,13]. A large Japanese claim-based study involving 718 patients with GPP and 27,773 patients with plaque psoriasis found a slight female preponderance in patients with GPP (51.5%) compared with those with plaque psoriasis, in which a minority of patients were female (38.7%) [13].

In the study, we found that more than 80% of pustular psoriasis was PPP, while GPP and ACH occurred more rarely. This finding is consistent with a large multi-cohort study by Twelves S et al. in which PPP accounted for 560 out of 863 (65%) patients, whereas GPP and ACH were much rarer [14]. In contrast, some Asian studies including those from Tosukhowong T et al. reported that GPP was more common than localized pustular psoriasis including PPP [10,11,12]. Ethnicity and genetics, but also different categorizations of pustular psoriasis, may contribute to explain the different prevalence between the GPP and PPP subtypes. We found that 27% of patients with pustular psoriasis also had concomitant PsA, which is a similar prevalence to PsA in patients with plaque psoriasis [15]. This finding is interesting considering that there are few data in the literature on the matter and is consistent with the finding of Ohata C et al. in Japanese patients with GPP (24 out of 102, i.e., 23%) [16]. Of note, the anatomical proximity between the nail apparatus and distal interphalangeal joints supports such an association in ACH [6]. We found a higher prevalence of tobacco smoking in patients with PPP, reaching a prevalence of 70%, as expected [6]. Local and systemic exposure to smoke may trigger pustular psoriasis in patients with a predisposed background through the excretion of stress-induced cytokines in keratinocytes as a Koebner phenomenon [14].

In our study, the proportion of concomitant plaque psoriasis was 31% in GPP, 20% in PPP, and 18% in ACH, which is consistent with Twelves S et al. [17]. Pustular psoriasis is a rare disease whose incidence and prevalence vary across geographical region and ethnicities. Interpreting the available data is challenging because of several issues including misdiagnosis, lack of awareness, different data-sources, and the low number of patients [18,19]. As an example, different definitions of pustular psoriasis can be used across studies as well as the inclusion (or lack of differentiation from) palmoplantar pustulosis. In other cases, data from hospital audits or claims database analyses have not been extrapolated to reflect the national population-level prevalence estimates [20]. A French study with a questionnaire-based assessment, estimated a disease occurrence of 1.76 cases per million [21]. Another Japanese study reported a GPP case estimate of 7.46 patients per million, with 2.87 patients per million presenting with acute GPP [22]. A review of a national insurance claims database in the Republic of Korea conducted between 2011 and 2015 estimated a prevalence of 1.2 cases per 10,000 persons. More interestingly, in more than 200,000 patients with psoriasis, 2.0–2.7% were classified as having GPP [23].

Since the first description of GPP by Leopold von Zumbush in 1910, several descriptions and diagnostic criteria have been developed, up to the ERASPEN criteria. In contrast to plaque psoriasis, in pustular psoriasis, the interleukin (IL)-36 pathway appears to play a pivotal role in the pathogenesis [2]. The recognition of loss-of-function mutations in the IL-36 receptor antagonist gene (IL36RN) and the overexpression of IL-36 cytokines in skin lesions supports the central role of IL-36. Recently, other mutations (i.e., variants of *CARD14*, encoding a keratinocyte adaptor protein, and *AP1S3*, which encodes a subunit of the adaptor protein 1 complex) have been identified in patients with GPP [1,2,5]. Transcriptome analysis of GPP lesions has shown an increase in innate immune inflammation response over TH1/TH17-related transcripts including IL-1β, IL-36α, IL-36γ, and enriched neutrophil and monocyte transcripts such as CXCL1, CXCL2, and CXCL8 [24]. The emerging development of specific inhibitors of IL-36R signaling (i.e., spesolimab and imisolimab) was followed by very encouraging results in terms of safety and efficacy in clinical trials [25]. Recent PPP trials are also focusing on the blockage of IL-36. Despite these advantages, there are still many important knowledge gaps, and further epidemiological studies and national registries are needed to characterize pustular psoriasis. The systematic genetic characterization of the patients may identify additional genetic variants that could provide effective therapeutic targets [26].

We acknowledge some limitations of the study. First, a potential selection bias in the source of the patients may have caused the underreporting of mild forms of plaque psoriasis because they were not referred to the hospital. We also did not test for the presence of genetic mutations (i.e., IL36RN, CARD14, and AP1S). Finally, we did not extrapolate our results to reflect the national population-level estimates. Nonetheless, this study has some strengths including the fact that all patients were visited by us, by the rheumatologist, and when needed, we also performed a biopsy for diagnostic confirmation over a period of seventeen years.

## 6. Conclusions

In conclusion, this study contributes to provide up-to-dated insights into the clinical characteristics of patients with pustular psoriasis in Italy. As with many rare diseases, accurately describing the epidemiology of pustular psoriasis is challenging and further studies are needed.

## Figures and Tables

**Table 1:** The main characteristics of patients with pustular psoriasis (*n* = 140).

	Palmoplantar Pustular Psoriasis (*n* = 116)	Acrodermatitis Continua of Hallopeau (*n* = 11)	Generalized Pustular Psoriasis (*n* = 13)	*p* *
Female gender, *n* (%)	75 (65)	8 (73)	8 (62)	0.884
Age, median (interquartile range)	58 (43–65)	57 (54–68)	59 (39–70)	0.918
Smoking, *n* (%)	82 (71)	4 (36)	5 (39)	0.008
Body mass index	26 (23–32)	24 (23–25)	24 (22–26)	0.001
Concomitant plaque psoriasis, *n* (%)	23 (20)	2 (18)	4 (31)	0.597
Psoriatic arthritis, *n* (%)	33 (28)	4 (36)	1 (8)	0.198
Palmar involvement only, *n* (%)	9 (8)	9 (82)	0	NA
Plantar involvement only, *n* (%)	39 (33)	2 (18)	0	NA
Both palmar and plantar, *n* (%)	68 (59)	0	0	NA
Inverse psoriasis, *n* (%)	5 (4)	0	0	NA

* Fisher’s exact test and the Kruskal–Wallis test for the categorical and unpaired non-normally distributed quantitative data, respectively.

## Data Availability

Data available on request from corresponding Author.
